# Unraveling the mystery of the glacier bear: Genetic population structure of black bears (*Ursus americanus*) within the range of a rare pelage type

**DOI:** 10.1002/ece3.6490

**Published:** 2020-06-27

**Authors:** Tania Lewis, Gretchen Roffler, Anthony Crupi, Ramona Maraj, Neil Barten

**Affiliations:** ^1^ Glacier Bay National Park and Preserve Gustavus AK USA; ^2^ Division of Wildlife Conservation Alaska Department of Fish and Game Douglas AK USA; ^3^ Faculty of Environmental Design University of Calgary Calgary AB Canada; ^4^ Division of Wildlife Conservation Alaska Department of Fish and Game Dillingham AK USA

**Keywords:** biogeography, black bears, color morph, glacier bear, mammals, microsatellites, population structure

## Abstract

Glacier bears are a rare grey color morph of American black bear (*Ursus americanus*) found only in northern Southeast Alaska and a small portion of western Canada. We examine contemporary genetic population structure of black bears within the geographic extent of glacier bears and explore how this structure relates to pelage color and landscape features of a recently glaciated and highly fragmented landscape. We used existing radiocollar data to quantify black bear home‐range size within the geographic range of glacier bears. The mean home‐range size of female black bears in the study area was 13 km^2^ (*n* = 11), whereas the home range of a single male was 86.9 km^2^. We genotyped 284 bears using 21 microsatellites extracted from noninvasively collected hair as well as tissue samples from harvested bears. We found ten populations of black bears in the study area, including several new populations not previously identified, divided largely by geographic features such as glaciers and marine fjords. Glacier bears were assigned to four populations found on the north and east side of Lynn Canal and the north and west side of Glacier Bay with a curious absence in the nonglaciated peninsula between. Lack of genetic relatedness and geographic continuity between black bear populations containing glacier bears suggest a possible unsampled population or an association with ice fields. Further investigation is needed to determine the genetic basis and the adaptive and evolutionary significance of the glacier bear color morph to help focus black bear conservation management to maximize and preserve genetic diversity.

## Graphical abstract

We examine contemporary genetic population structure of black bears within the geographic extent of a rare color morph known as glacier bears. We explore how this structure relates to pelage color and landscape features of a recently glaciated and highly fragmented landscape of Southeast Alaska and western Canada. This research is the first to be done on the biogeography of this rare color morph of black bear. Our hope is that this manuscript will be the first step to understand and conserve glacier bears as well as an important step towards protecting the biodiversity of black bears in this region and the world.

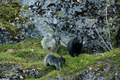

## 1 INTRODUCTION

Glacier advances and retreats during the late Quaternary period sculpted landscapes across the world and greatly influenced distribution of contemporary biota (Hewitt, 1996, 2000). Ice expansion contracted species' ranges, often reducing effective population sizes which over time increased differentiation between populations due to bottlenecks and genetic drift. As ice contracted, species' ranges often expanded and populations that had become differentiated by genetic isolation came into secondary contact with each other (Hewitt, 1996; Petit, Aguinagalde, & de Beaulieu, 2003). In some regions, such as the Southeast Alaska (SEAK) and western Canadian coast of North America, deep marine fjords left by the Pleistocene ice advances, steep rugged mountains from ongoing tectonism, and large glaciers and ice fields maintained by persistent cold precipitation from the Gulf of Alaska has created a fragmented landscape that inhibits mammalian dispersal (Klein, 1965; Mann, 1986; Mann & Hamilton, 1995). With the exception of possible coastal refugium, the entirety of SEAK was covered in ice at the last glacial maximum approximately 18,000 years ago (Carrara, Ager, & Baichtal, 2007; Cook, Dawson, & MacDonald, 2006; Heaton, Talbot, & Shields, 1996; Klein, 1965). Consequently, the relationship between landscape and species diversity is measurable because species have undergone drastic contraction and subsequent expansion of ranges in a relatively short period of time and the landscape is naturally fragmented yet largely free from human influence (Dawson, MacDonald, & Cook, 2007; Lewis, Pyare, & Hundertmark, 2015; Peacock, Peacock, & Titus, 2007; Sawyer, MacDonald, Lessa, & Cook, 2019). In SEAK, past landscape fragmentation from glaciation led to the divergence of ancient lineages of multiple mammalian species including dusky shrews (*Sorex monticolus*; Demboski & Cook, 2001), American marten (*Martes americanus*; Stone, Flynn, & Cook, 2002; Dawson et al., 2017), mountain goats (*Oreamnos americanus*; Shafer, Cullingham, Cete, & Coltman, 2010), wolves (*Canis lupus*; Weckworth, Talbot, Sage, Person, & Cook, 2005), brown bears (*Ursus arctos*; Talbot & Shields, 1996; Paetkau, Shields, & Strombeck, 1998; Lewis et al., 2015), and black bears (*Ursus americanus;* Byun, Koop, & Reimchen, 1997; Stone & Cook, 2000; Peacock et al., 2007; Puckett, Etter, Johnson, & Eggert, 2015) that are now converging in this secondary contact zone. Some areas in northern SEAK, such as Glacier Bay, were also glaciated during the Little Ice Age approximately 280 years ago followed by the most rapid ice retreat recorded in history (Connor, Streveler, Post, Monteith, & Howell, 2009; Lawrence, 1958), thus providing an even shorter time frame from which to compare landscape change with biodiversity. Since the retreat of the most recent ice age, the shoreline of Glacier Bay has been colonized by multiple terrestrial mammal species, including black bears.

In addition to landscape features, species' life‐history traits influence their dispersal capability and genetic connectivity. Black bears are large vagile mammals with highly variable home‐range sizes in North America ranging from 4.8 to 41 km^2^ for females and 6.1 to 173 km^2^ for males (Powell, Zimmerman, & Seaman, 1997). Males generally disperse outside of their natal range whereas females stay within the range of their mothers (Schwartz & Franzmann, 1992). Little is known about the home‐range size of black bears on the mainland in SEAK. Black bears ranging from Glacier Bay to Yakutat Bay have been designated into subspecies *Ursus americanus emmonsii*, while most other black bears in SEAK are placed in the subspecies *Ursus americanus pugnax*, with some question as to the validity of these subspecies designations (MacDonald & Cook, 2009). Molecular evidence based on analyses of mitochondrial DNA suggests two distinct lineages of black bears that diverged over one million years ago and converge today south of Juneau, Alaska (Byun et al., 1997; Puckett et al., 2015; Stone & Cook, 2000). Using nuclear DNA, Peacock et al. (2007) also found clear evidence of these two ancient black bear lineages (Mainland and Island clusters), which suggests that admixture of the two lineages after secondary contact has been limited. The authors hypothesize that barriers to movement such as ice fields and fjords maintain genetic differentiation among black bear populations in SEAK. In previous studies of black bear genetic relatedness in SEAK (Peacock et al., 2007), genetic samples were derived largely from hunter‐harvested bears so large protected areas such as Glacier Bay National Park were excluded from sampling due to hunting restrictions. Further sampling in protected areas will allow us to answer questions regarding putative barriers to gene flow such as ice fields and fjords.

**FIGURE 1:**
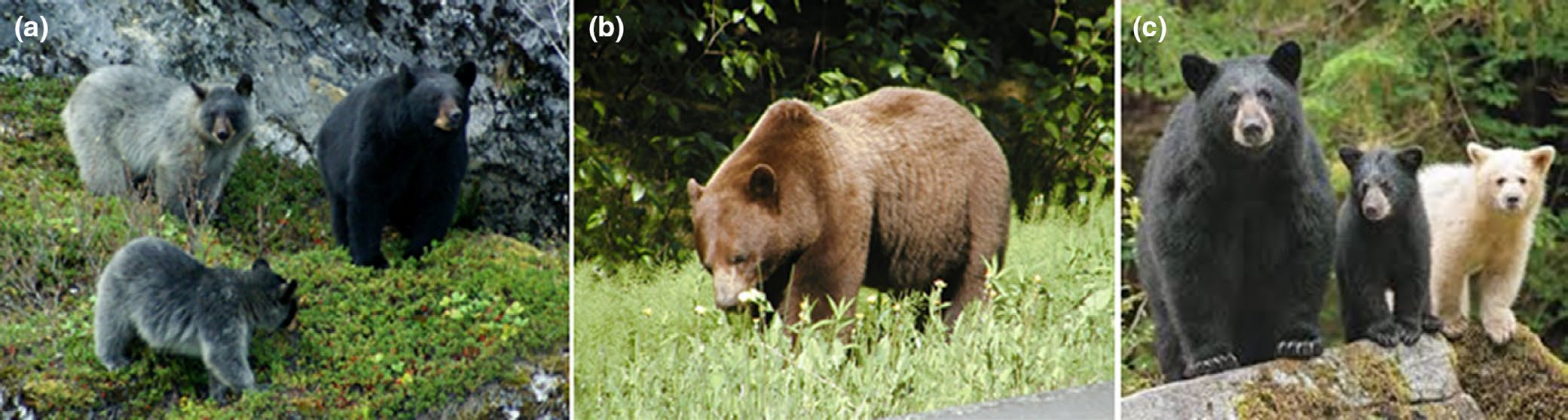
Photographs showing the variety of color morphs of black bears in the Pacific Northwest including glacier (a: two cubs on left), black (a: adult bear on right) and brown morphs (b) from Glacier Bay Alaska (C. Edwards and T. Lewis/NPS Photographs), and black (c: adult and cub on left) and Kermode morph (c: cub on right) from the Great Bear Rainforest in British Columbia (Ian McAllister in the Times Colonist 15 February 2019)

**FIGURE 2:**
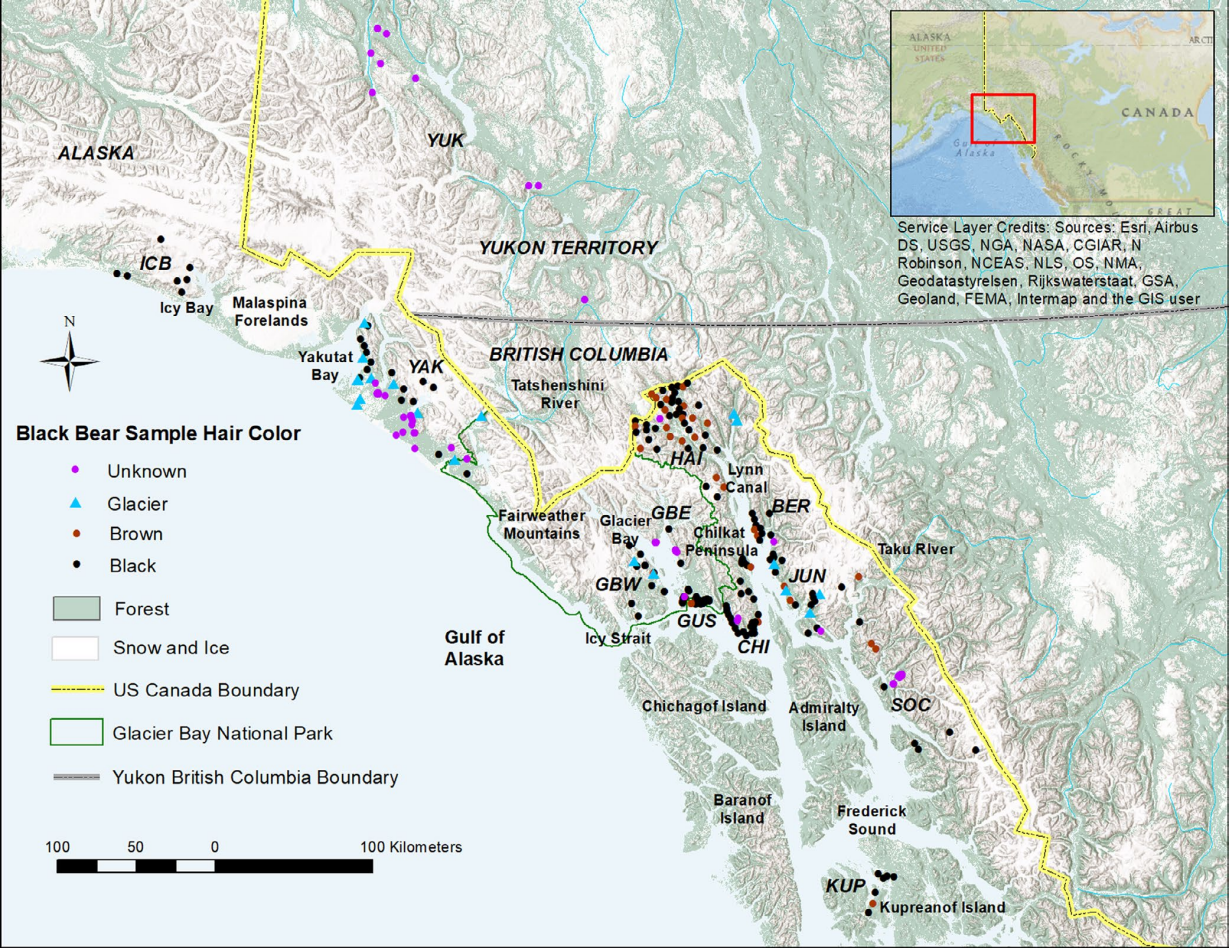
Sample locations of 284 black bears by color phase and sampling region including: Icy Bay (ICB), Yakutat (YAK), Glacier Bay West (GBW), Glacier Bay East (GBE), Gustavus (GUS), Chilkat Peninsula (CHI), Haines (HAI), Yukon (YUK), Berners (BER), Juneau (JUN), South Coast Mainland (SOC), and Kupreanof Island (KUP) in Southeast Alaska and Yukon Territory, 2002–2014. Note that these are general locations. AS 16.05.815(d) mandates confidentiality of personal information contained in fish and wildlife harvest and usage data; telemetry radio frequencies of monitored species; denning sites; raptor nest locations; the specific location of animal capture sites used for wildlife research or management; and the specific location of fish and wildlife species

Black bears exhibit a variety of pelage colors but are predominantly black throughout their North American range with some exceptions including cinnamon/brown color morphs found most commonly in the arid regions of the western continent, white morphs (Kermode or “spirit” bears) found in a small portion of southern coastal British Columbia (BC) Canada, and a rare gray/blue morph (glacier bears) found in northern SEAK and BC (Figure 1; Rounds, 1987). Glacier bears' pelage ranges from white to black with silver‐tipped guard hairs, and black mothers can produce glacier offspring, and vice versa, indicating polygenic, epigenetic regulation and/or a recessive mutation. Ritland, Newton, and Marshall (2001) found the Kermode color morph to be caused by a single‐nucleotide substitution that caused an amino acid change in the melanocortin 1 receptor (Mc1r) locus. This receptor responds to levels of melanocyte‐stimulating hormone to regulate pigment production and is recessive, so only animals homozygous at this locus express the Kermode color morph whereas heterozygotes may act as a reservoir for the gene in the population. The genetic basis of the glacier color morph is, however, unknown. Glacier bears have been observed from Yakutat Bay to the Taku River (Figure 2; Lewis, Stanek, & Young, 2020). In SEAK, the relative frequency of the color morphs can be inferred from harvest record from 1990 to 2018 during which time 96.6% of the black bears harvested were black, 3% cinnamon, and 0.4% glacier (ADF&G, 2020). Cinnamon‐colored black bears are present on the shoreline of Lynn Canal to the northeast of Glacier Bay (Hessing, 2005), whereas glacier bears appear to be the most abundant on the west side of Glacier Bay and north on the Yakutat forelands where cinnamon‐colored bears are very rare (Barten, 2005). The first cinnamon‐colored black bear was reported on the shoreline of Glacier Bay in 1967 (Home, 1973), and they are now common on the lower eastern portion of the bay (T. Lewis personal observation). If color morph is indicative of subspecies (glacier bears signifying *U. a. emmonsii* and cinnamon‐colored bears signifying *U. a. pugnax*), the two subspecies may overlap within Glacier Bay National Park and northern SEAK (MacDonald & Cook, 2009).

The genetic basis for the glacier pelage and frequency in black bear populations are unknown, making it difficult to manage the rare color morph to conserve the genotypic and phenotypic diversity of black bears in this region. Although glacier bears are protected from legal harvest in the portion of their range encompassed by Glacier Bay National Park, they are legally hunted in the large majority of their range. Sport hunters and guides specifically target glacier bears, particularly around Yakutat AK. Directed harvest may decrease the number of bears carrying the glacier genotype and lead to a decrease in the phenotype and likely associated genetic diversity. Information gained in this research will provide background for assessing the vulnerability and conservation concern for genotypic and phenotypic diversity of black bears in SEAK and western Canada.

We sought to begin to unravel the mystery surrounding the rare glacier bear color morph with the following objectives: (a) determine home‐range size of black bears in northern SEAK with Global Position System (GPS) collar data; (b) use microsatellites to identify the number and geographic extent of black bear populations within the range of glacier bears and assess the number of migrants between sampling regions; (c) identify landscape features that promote or limit genetic connectivity between black bear populations; and (d) explore the relationship between pelage color and population structure.

## 2 MATERIALS AND METHODS

### 2.1 Study area

The study area comprises approximately 110,000 km^2^ of northern SEAK, extending from Icy Bay along the Gulf of Alaska in the United States, and northwest BC and southwest Yukon Territory of Canada, to Kupreanof Island in SEAK (Figure 2). The study area does not include Admiralty, Baranof, or Chichagof islands because black bears do not inhabit these islands. The climate of this region varies between cool summers and wet winters in SEAK and BC to extreme temperatures and dry conditions in the Yukon Territory. Topography varies with rugged mountains rising to 4,631 m elevation, ice fields descending to tidewater glaciers at sea level, and includes broad glacially carved river valleys, interior plateaus, marine fjords, and islands.

### 2.2 Home‐range size

Between 2003 and 2015, the Alaska Department of Fish and Game (ADF&G) investigated black bear habitat use and movement patterns in northern SEAK to inform research and management objectives. Black bears were captured using culvert traps, modified Aldrich foot and bucket snares, and free‐range darting techniques. Bears were anesthetized with tiletamine hydrochloride and zolazepam hydrochloride (Telazol®, Fort Dodge Animal Health) at a dosage of 7–10 mg/kg estimated body weight (Taylor, Reynolds, & Ballard, 1989). All animal capture protocols were approved by ADF&G's Division of Wildlife Conservation Institutional Animal Care and Use Committee protocol 2003‐0010 and conformed to the procedures outlined by the American Society of Mammalogists (Sikes & Gannon, 2011). Each animal was marked with a GPS radiocollar (Telonics, Inc.) equipped with a release mechanism and programmed to collect locations at regular intervals (30–60 min or daily) during the period when bears are typically active in SEAK (15 April–1 November). A vestigial premolar tooth was collected to determine bear age based on cementum annuli (Matson et al., 1993), and the resulting age data were archived in ADF&G's harvest database (ADF&G, 2020). We estimated individual animal composite home ranges of twelve black bears from GPS locations using the Geospatial Modeling Environment (Beyer, 2015) and ArcGIS (version 10.5; ESRI). We generated a fixed kernel density estimate (Worton, 1989) with a 95% utilization distribution using a least‐squares cross‐validation bandwidth estimator (Horne & Garton, 2006; Seaman & Powell, 1996). We truncated home ranges that extended into ocean habitats by clipping the polygon to the defined shoreline. Means are presented ±1 standard deviation.

### 2.3 Genetic sample collection

From 2002 to 2014, we collected black bear tissue samples from harvested bears and bears captured for research purposes, as well as noninvasive hair samples from rub trees, hair snares, and scented hair traps from multiple research projects (Crupi, Waite, Flynn, & Beier, 2017; Lewis et al., 2015; Partridge, Smith, & Lewis, 2009). At the time of collection, we recorded hair color for most samples as black, glacier, or brown/cinnamon/chocolate (grouped as brown). Samples without pelage color information were categorized as unknown. Capture locations were recorded using GPS, and harvest locations were reported by hunters as “specific locations” and later digitized in ArcGIS. Samples were grouped into 12 distinct sampling regions separated by marine waters and/or mountain ranges from northwest to southeast: Icy Bay, Yakutat, Glacier Bay West (GBW), Glacier Bay East (GBE), Gustavus, Chilkat Peninsula, Haines, Yukon, Berners, Juneau, South Coast Mainland, and Kupreanof Island (Figure 2).

### 2.4 Genotyping

Wildlife Genetics International (Nelson BC) extracted DNA with Qiagen's DNeasy tissue kits (Qiagen) and amplified and genotyped DNA as described in Paetkau et al. (1998). Sequence‐based analysis of a portion of the 16S rRNA mitochondrial gene was used to confirm species (Johnson & O'Brien, 1996; Pongracz, Paetkau, Branigan, & Richardson, 2017). Individual black bears were identified using seven microsatellite markers selected for high relative variability in the study populations, including G10B, G1D, G10J, G10M, G10U, Mu50, Mu59, (Paetkau et al., 1998; Taberlet et al., 1997) plus one marker to determine sex (unpublished ZFX/ZFY primer pair designed by Wildlife Genetics International). Computerized comparisons of all pairs of unique genotypes were used to reduce genotyping errors from allelic dropout in accordance with Paetkau (2003) and Kendall et al. (2009). Microsatellite genotyping of each individual black bear was extended to include REN145PO7, G10C, CXX20, G10H, MSUT2, G10P, G1A, CPH9, CXX110, MU23, G10L, MU26, D123, and D1a (Breen et al., 2001; Kitahara, Isagi, Ishibashis, & Saitoh, 2000; Meredith, Rodzen, Banks, & Jones, 2009; Paetkau et al., 1998; Taberlet et al., 1997). Although microsatellites are less accurate than a large single‐nucleotide polymorphism marker panel for detecting admixture or recent migrants (Puckett & Eggert, 2016), the panel of 21 microsatellites was highly variable (Paetkau et al., 1998) and thus informative.

### 2.5 Genetic variation

We tested for deviation from Hardy–Weinberg proportions (HWP) and linkage disequilibrium (LD) within the black bear sampling regions and between pairs of loci using GENEPOP (Raymond & Rousset, 1995) with the Markov chain Monte Carlo (MCMC) approximation of Fisher's exact test and a simulated exact test, respectively. We ran 10,000 dememorizations, 100 batches, and 5,000 iterations and applied a Bonferroni‐corrected alpha level of 0.05 for multiple comparisons. We used FSTAT v.2.9.3 (Goudet, 2001) to measure the level of genetic diversity and variation by calculating the mean number of alleles per locus (*A*), number of private alleles (*PA*), rarefied allelic richness (*A_R_*) corrected for sample size differences using hp‐rare 1.0 (Kalinowski, 2005), and mean observed and expected heterozygosities (*H_o_* and *H_e_*). We also tested whether inbreeding coefficients (*F_IS_*) were significantly different from zero with 5,000 randomizations using FSTAT.

### 2.6 Population structure

To assess black bear population structure, we used two individual‐based Bayesian clustering approaches: STRUCTURE v2.3.3 (Pritchard, Stephens, & Donnelly, 2000) and GENELAND 3.3.0 (Guillot, Mortier, & Estoup, 2005). In STRUCTURE, we performed analyses both including and excluding the sampling location as a prior. Sampling regions were determined as clusters of sample locations separated from each other by ocean or mountains. We used an admixture model, defined allele frequencies as correlated due to suspected shared ancestry and migration between populations and performed MCMC simulations of 1,000,000 iterations with a burn‐in period of 100,000. We performed 10 repetitions per simulation to test for values of *K* (genetic clusters) from 1 to 20. Because sample sizes were uneven across sampling locations, we determined *K* using the MedMeaK, MaxMeaK, MedMedK, and MaxMedK estimators (Puechmaille, 2016) using StructureSelector (Li & Liu, 2018). The maximum value of *K* over the 10 repetitions was determined by assigning a sampling location to a cluster if the mean or median membership coefficient ≥0.5. We calculated the number of individuals of different pelage color (black, brown, and glacier) by assigned population. We calculated population pairwise multilocus estimates of *F*_ST_ (Weir & Cockerham, 1984) using GENEPOP and Jost's *D*, which measures genetic differentiation using differences in true allelic diversity correcting for sampling bias, (Jost, 2008) using GenAlEx 6.5 (Peakall & Smouse, 2006). Ten thousand random permutations were used to test significance of pairwise *F*_ST_ and Jost's *D*.

GENELAND also assigns individuals to genetic clusters but incorporates spatial information from individual sample locations. We tested for values of *K* from 1 to 20, using 500,000 iterations with a burn‐in period of 20,000. We defined allele frequencies as correlated as well as uncorrelated based on Dirichlet distributions (Pritchard et al., 2000) and assessed the influence of different values of spatial uncertainty (between 1,000 and 10,000 m) on population structure, replicating each model 20 times and ranking them by the mean logarithm of posterior probability.

We also inferred black bear genetic structure by clustering individual genotypes using a discriminant analysis of principal components (DAPC, Jombart, Devillard, & Balloux, 2010) with 11 discriminant analysis axes retained using the R package adegenet 2.0.1 (Jombart, 2008). Because this approach can detect genetic clusters even in cases of hierarchical structure or isolation by distance, it is more appropriate than Bayesian methods in the presence of complex genetic spatial structure (Jombart et al., 2010).

We tested for a genetic signature of isolation by distance using Mantel tests of pairwise geographic versus genetic distance between individuals performing 99,999 random permutations in GenAlEx 6.4 (Peakall & Smouse, 2006).

### 2.7 Detecting recent migrants

To estimate recent migration, we used Bayesian individual assignment (Rannala & Mountain, 1997) implemented in GeneClass 2.0 (Piry et al., 2004) based on sampling location. We used a probability threshold of 0.01, and a MCMC probability computation with 100,000 simulated individuals. We detected first‐generation migrants by estimating the ratio of *L_HOME_/L_MAX_*, where *L_HOME_* is the likelihood of detecting an individual in the population where it was sampled, and *L_MAX_* is the likelihood of detecting that individual in all sampled populations (Paetkau, Slade, Burden, & Estoup, 2004).

## 3 RESULTS

### 3.1 Home‐range size

We captured and radiocollared one male and 11 female black bears between Juneau and Yakutat, AK. The male black bear was 6 years old and occupied an 86.9 km^2^ home range, nearly three times larger than any female home range, whereas mean female home‐range size was 13.0 km^2^ (range 5.4–30.5 km^2^; Table S1). Age and reproductive status of the female bears varied as mean female age was 9.6 ± 5.7 years (range 3–19 years), and three of the animals supported dependent offspring. Four of the female black bears were transported 8–40 km from their capture location, and all returned to their home range within 4–25 days of relocation. Home ranges for these animals were estimated only from the locations collected subsequent to their return.

### 3.2 Genetic variation

We successfully genotyped 284 black bears from 12 sampling regions (Figure 2): 114 from noninvasive hair samples (39 female and 75 male) and 170 from tissue samples of harvested animals (36 female and 134 male) totaling 75 females and 209 males for a male‐biased sex ratio of 2.79 males per female. Sample size was variable across regions with the highest number of samples from Yakutat (*n* = 52) and the lowest from Kupreanof (*n* = 7; Table S2). No significant deviations from HWP occurred in any loci or sampling region after adjusting for multiple tests. Six of 2,520 comparisons (Bonferroni correction) between pairs of loci displayed significant LD in four black bear sampling regions (Berners, GBE, Haines, Yakutat), but there was no consistent pattern or repetition among sampling regions, and none of the locus pairs across all sampling regions were significant; thus, all loci were retained for subsequent analyses. All loci were polymorphic, with 2–11 alleles across loci (*A* = 5.26). *PA* was highest in the far northern (Icy Bay) and southern (South Coast and Kupreanof) sampling regions. Measures of genetic diversity varied across sampling regions with highest levels of *A_R_* (range = 3.29–5.29), *H_o_* (range = 0.586–0.798), and *H_e_* (range = 0.556–0.762) in the Juneau, South Coast, and Yukon sampling region (Table S2). Inbreeding coefficients (*F*_IS_) were not significantly different from zero.

### 3.3 Population structure

**FIGURE 3:**
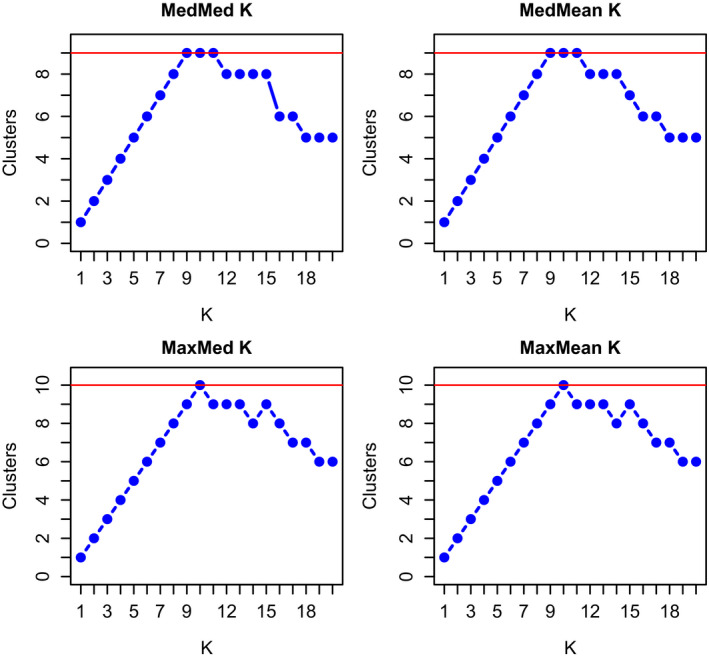
Estimated number of genetic clusters (*K*) using median of means (MedMeaK), maximum of means (MaxMeaK), median of medians (MedMedK), and maximum of medians (MaxMedK) in STRUCTURE

**FIGURE 4:**
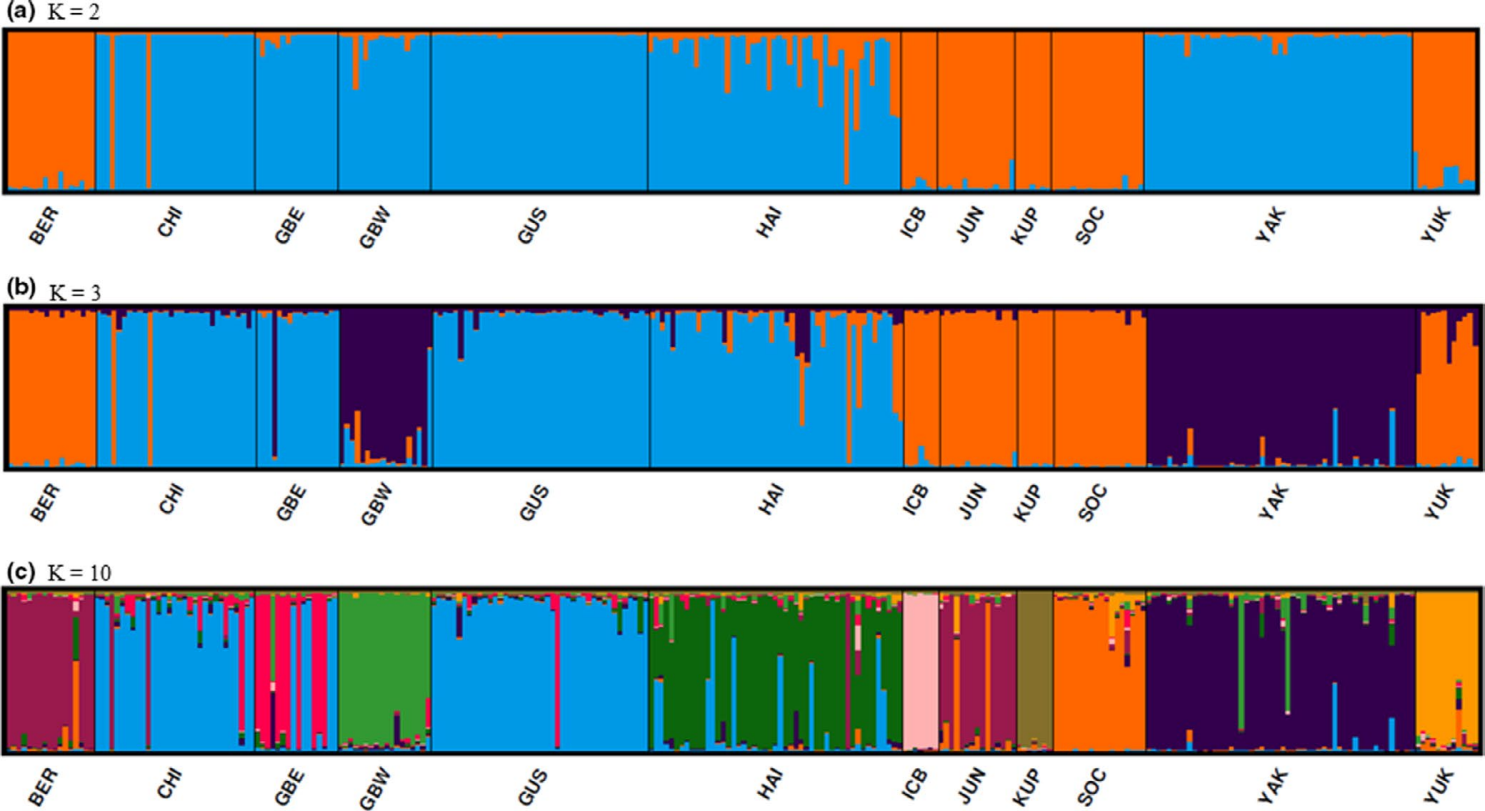
(a, b, and c) STRUCTURE output for 21 loci genotypes of 284 black bears representing estimated ancestry in each identified population at *K* = 2, *K* = 3, and *K* = 10 in SEAK and Yukon Territory, 2002–2014. Sampling regions are as follows: Berners (BER), Chilkat Peninsula (CHI), Glacier Bay East (GBE), Glacier Bay West (GBW), Gustavus (GUS) Haines (HAI), Icy Bay (ICB), Juneau (JUN), Kupreanof Island (KUP), South Coast Mainland (SOC), Yakutat (YAK), and Yukon (YUK)

The best supported model from STRUCTURE indicates *K* = 10. The MedMeaK, MaxMeaK, MedMedK, and MaxMedK estimators detected 10 populations both with and without a sampling location prior (Figure 3). In this model, the GBE, GBW, Haines, Icy Bay, Kupreanof, South Coast, Yakutat, and Yukon populations formed their own distinct clusters, Berners and Juneau clustered together, and Chilkat and Gustavus clustered together (Figure 4c). Sampling regions Icy Bay and GBW had the highest proportion of ancestry assigned to their own population (0.966 and 0.908, respectively) whereas GBE and Kupreanof had the lowest (0.565 and 0.675, respectively; Table 1). Bears sampled in GBE shared 31% of their ancestry with adjacent Chilkat/Gustavus population, whereas bears from Kupreanof at the southern end of the study area shared 27% of their ancestry with Icy Bay at the northern end of the study area.

**TABLE 1:** Mean proportion of ancestry of black bears by 12 sampling regions (rows) assigned to 10 populations (columns) by program STRUCTURE *K* = 10 in Southeast Alaska and Yukon Territory, 2002–2014

Sampling region	Assigned population									
	YAK	BER/JUN	ICB	CHI/GUS	SOC	GBE	YUK	KUP	GBW	HAI
BER	0.009	0.856	0.008	0.009	0.056	0.009	0.007	0.005	0.008	0.034
CHI	0.015	0.066	0.003	0.782	0.004	0.058	0.005	0.032	0.016	0.019
GBE	0.030	0.005	0.008	0.309	0.003	0.565	0.005	0.018	0.039	0.019
GBW	0.025	0.006	0.004	0.016	0.004	0.017	0.007	0.006	0.908	0.007
GUS	0.014	0.003	0.003	0.788	0.004	0.037	0.029	0.097	0.008	0.017
HAI	0.018	0.031	0.009	0.122	0.008	0.019	0.011	0.012	0.024	0.746
ICB	0.005	0.004	0.966	0.004	0.002	0.003	0.004	0.004	0.004	0.004
JUN	0.012	0.774	0.006	0.007	0.140	0.006	0.024	0.006	0.013	0.010
KUP	0.003	0.005	0.267	0.004	0.027	0.006	0.004	0.675	0.006	0.004
SOC	0.010	0.016	0.008	0.008	0.885	0.011	0.041	0.007	0.008	0.006
YAK	0.876	0.007	0.006	0.020	0.006	0.006	0.009	0.008	0.050	0.012
YUK	0.017	0.012	0.012	0.007	0.114	0.008	0.800	0.005	0.013	0.012

Note

Populations are as follows: Berners/Juneau (BER/JUN), Chilkat Peninsula/Gustavus (CHI/GUS), Glacier Bay East (GBE), Glacier Bay West (GBW), Haines (HAI), Icy Bay (ICB), Kupreanof Island (KUP), South Coast Mainland (SOC), Yakutat (YAK), and Yukon (YUK).

Results from GENELAND indicated *K* = 7 for both correlated and uncorrelated allele frequencies and both 5 km and 10 km spatial uncertainty. These results clustered the STRUCTURE *K* = 10 populations GBE with Chilkat/Gustavus, Yakutat with GBW, and Yukon with South Coast.

**FIGURE 5:**
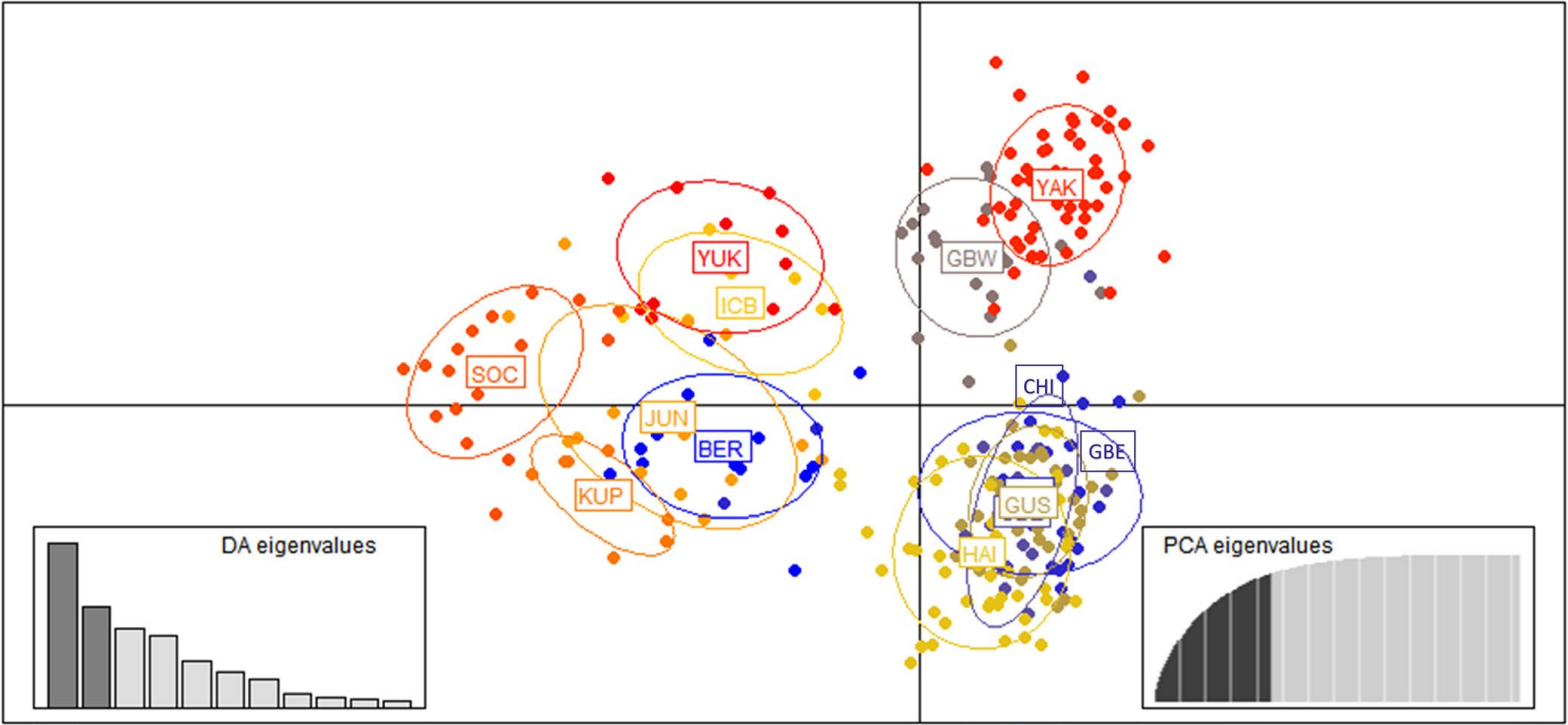
Results of discriminant analysis of principal components (DAPC) with number of DA axes retained = 11, for 284 black bears in 12 sampling regions in Southeast Alaska and Yukon Territory, 2002–2014. Sampling regions are as follows: Berners (BER), Chilkat Peninsula (CHI), Glacier Bay East (GBE), Glacier Bay West (GBW), Gustavus (GUS), Haines (HAI), Icy Bay (ICB), Juneau (JUN), Kupreanof Island (KUP), South Coast Mainland (SOC), Yakutat (YAK), and Yukon (YUK)

DAPC results group Yakutat and GBW in the upper right quadrant; Chilkat, GBE, Gustavus, and Haines in the lower right quadrant; and the rest of the sampling regions left of the center axis (Figure 5). South Coast, Yakutat, and GBW show the least overlap with other groups indicating more distinct genetic clusters compared to the rest.

We found a significant signature of isolation by distance (*R* ^2^ = 0.411, *p* = .0001) in the Mantel test of geographic versus genetic distance. Pairwise comparisons of *F*_ST_ and Jost's *D* results at *K* = 10 indicate that all populations were significantly differentiated from each other (Table 2). Kupreanof and Icy Bay populations had the highest differentiation from other populations, whereas the least differentiation was seen between Chilkat/Gustavus to Haines and GBE, and South Coast to Yukon.

**TABLE 2:** Pairwise comparison of *F*_ST_ (below the diagonal) and Jost's *D* values (above the diagonal) from analyses among black bear populations based on STRUCTURE *K* = 10 in Southeast Alaska and Yukon Territory, 2002–2014

	BER/JUN	CHI/GUS	GBE	GBW	HAI	ICB	KUP	SOC	YAK	YUK
BER/JUN		0.374	0.404	0.449	0.352	0.498	0.548	0.361	0.425	0.439
CHI/GUS	0.090		0.208	0.266	0.185	0.484	0.575	0.504	0.257	0.400
GBE	0.094	0.057		0.345	0.259	0.524	0.542	0.472	0.361	0.444
GBW	0.103	0.071	0.090		0.288	0.528	0.490	0.465	0.210	0.306
HAI	0.079	0.048	0.065	0.071		0.477	0.528	0.430	0.333	0.345
ICB	0.116	0.126	0.137	0.136	0.116		0.668	0.592	0.553	0.432
KUP	0.126	0.146	0.140	0.126	0.126	0.174		0.532	0.634	0.598
SOC	0.069	0.107	0.096	0.095	0.085	0.119	0.107		0.513	0.283
YAK	0.107	0.073	0.102	0.061	0.088	0.152	0.169	0.116		0.350
YUK	0.086	0.091	0.096	0.069	0.073	0.096	0.127	0.048	0.087	

Note

All values were significant at *p* > .01. Populations are as follows: Berners/Juneau (BER/JUN), Chilkat Peninsula/Gustavus (CHI/GUS), Glacier Bay East (GBE), Glacier Bay West (GBW), Haines (HAI), Icy Bay (ICB), Kupreanof Island (KUP), South Coast Mainland (SOC), Yakutat (YAK), and Yukon (YUK).

### 3.4 Migrants

The sampling regions with the highest proportions of first‐generation migrants were Juneau (47% of individuals originating from Berners and 13% of individuals originating from South Coast) and GBE (31% of individuals originating from Gustavus, 6% of individuals originating from Chilkat, and 6% of individuals originating from Yakutat; Table 3). The two sampling regions on the northern (Icy Bay and Yukon) and southern ends of the study area (Kupreanof) did not have any recent migrants from any of the other sampling region.

**TABLE 3:** Proportion of first‐generation migrants using Bayesian individual assignment in GeneClass 2.0 within each sampling region of black bears, Southeast Alaska and Yukon Territory, 2002–2014

Sampling region	*n*	BER	CHI	GBE	GBW	GUS	HAI	ICB	JUN	KUP	SOC	YAK	YUK
BER	17	**0.88**	0.00	0.00	0.00	0.00	0.00	0.00	0.12	0.00	0.00	0.00	0.00
CHI	31	0.03	**0.68**	0.03	0.00	0.23	0.00	0.00	0.03	0.00	0.00	0.00	0.00
GBE	16	0.00	0.06	**0.56**	0.00	0.31	0.00	0.00	0.00	0.00	0.00	0.06	0.00
GBW	18	0.00	0.00	0.06	**0.94**	0.00	0.00	0.00	0.00	0.00	0.00	0.00	0.00
GUS	42	0.00	0.17	0.02	0.00	**0.81**	0.00	0.00	0.00	0.00	0.00	0.00	0.00
HAI	49	0.02	0.10	0.02	0.00	0.04	**0.82**	0.00	0.00	0.00	0.00	0.00	0.00
ICB	7	0.00	0.00	0.00	0.00	0.00	0.00	**1.00**	0.00	0.00	0.00	0.00	0.00
JUN	15	0.47	0.00	0.00	0.00	0.00	0.00	0.00	**0.40**	0.00	0.13	0.00	0.00
KUP	7	0.00	0.00	0.00	0.00	0.00	0.00	0.00	0.00	**1.00**	0.00	0.00	0.00
SOC	18	0.00	0.00	0.00	0.00	0.00	0.00	0.00	0.06	0.00	**0.94**	0.00	0.00
YAK	52	0.00	0.00	0.00	0.02	0.00	0.00	0.00	0.00	0.00	0.00	**0.98**	0.00
YUK	12	0.00	0.00	0.00	0.00	0.00	0.00	0.00	0.00	0.00	0.00	0.00	**1.00**

Note

Values in bold are the proportions of individuals that assigned to their source sampling region. Sampling regions are as follows: Berners (BER), Chilkat Peninsula (CHI), Glacier Bay East (GBE), Glacier Bay West (GBW), Gustavus (GUS) Haines (HAI), Icy Bay (ICB), Juneau (JUN), Kupreanof Island (KUP), South Coast Mainland (SOC), Yakutat (YAK), and Yukon (YUK).

### 3.5 Population‐color morph

Of the 284 bears sampled, 166 black bears were classified as black pelage (133 M, 33 F), 32 brown (25 M, 7 F), 22 glacier (12 M, 10 F), and 64 unknown (39 M, 25 F; Figure 2; Table 4). Black individuals were found in all populations with known colors (color morph was not reported for any of the Yukon samples), whereas brown individuals were found in six populations (Berners/Juneau, Chilkat/Gustavus, South Coast, Kupreanof, GBW, and Haines). Glacier bears were assigned to four populations: Yakutat (14 of 50), Berners/Juneau (4 of 32), GBW (2 of 21) and Haines (2 of 41).

**TABLE 4:** Number of individual black bears of different color morphs by assigned population from program STRUCTURE at *K* = 10 in Southeast Alaska and Yukon Territory, 2002–2014

Population	Black	Brown	Glacier	Unknown	Total
BER/JUN	21	5	4	2	32
CHI/GUS	67	7	0	7	81
GBE	4	0	0	8	12
GBW	16	1	2	2	21
HAI	23	15	2	1	41
ICB	7	0	0	0	7
KUP	6	1	0	0	7
SOC	8	3	0	10	21
YAK	14	0	14	22	50
YUK	0	0	0	12	12
Grand total	166	32	22	64	284

Note

Populations are as follows: Berners/Juneau (BER/JUN), Chilkat Peninsula/Gustavus (CHI/GUS), Glacier Bay East (GBE), Glacier Bay West (GBW), Haines (HAI), Icy Bay (ICB), Kupreanof Island (KUP), South Coast Mainland (SOC), Yakutat (YAK), and Yukon (YUK).

## 4 DISCUSSION

This study builds on previous research examining genetic population structure of black bears in SEAK by adding noninvasively collected hair samples from a large geographic area (Glacier Bay and Kluane National Parks) where harvest is not allowed. We found several new populations not previously identified, including Icy Bay, Yukon, GBW, GBE, and Chilkat/Gustavus. Results of this study support previous studies showing that marine fjords impede genetic connectivity of black bears (Peacock et al., 2007) as well as brown bears (Lewis et al., 2015; Paetkau et al., 1998). Lynn Canal and Glacier Bay, both approximately 100 km long/10–18 km wide north–south fjords, appear to act as barriers to genetic connectivity as indicated by results from program STRUCTURE, GENELAND, and DAPC analysis. STRUCTURE *K* = 2 divided the samples into a cluster comprised of the sampling regions west of Lynn Canal (Chilkat, GBE, GBW, Gustavus, Haines, and Yakutat), and a cluster combining the sampling regions from eastern Lynn Canal to the southern extent of the study area (Berners, Juneau, South Coast, Kupreanof), as well as Yukon to the north and Icy Bay located on the northwest extent of the study area (Figure 4a). STRUCTURE *K* = 3, GENELAND, and DAPC all cluster together bears west of Glacier Bay (Yakutat and GBW), bears between Glacier Bay and Lynn Canal (GBE, Gustavus, and Chilkat) and bears east of Lynn Canal (Juneau and Berners) together, supporting evidence that long marine fjords act as a barrier to connectivity (Figure 4b). DAPC and STRUCTURE *K* = 3 results group Icy Bay, Kupreanof, South Coast, and Yukon with the Berners and Juneau cluster (Figure 5), whereas GENELAND separates each of these groups into three populations—Icy Bay, Kupreanof, and South Coast/Yukon. In addition to genetic differentiation found across marine fjord barriers, isolation by distance is also evident in DAPC results that indicate within each cluster, sampling regions close to each other geographically overlap, and those that are farther away do not.

**FIGURE 6:**
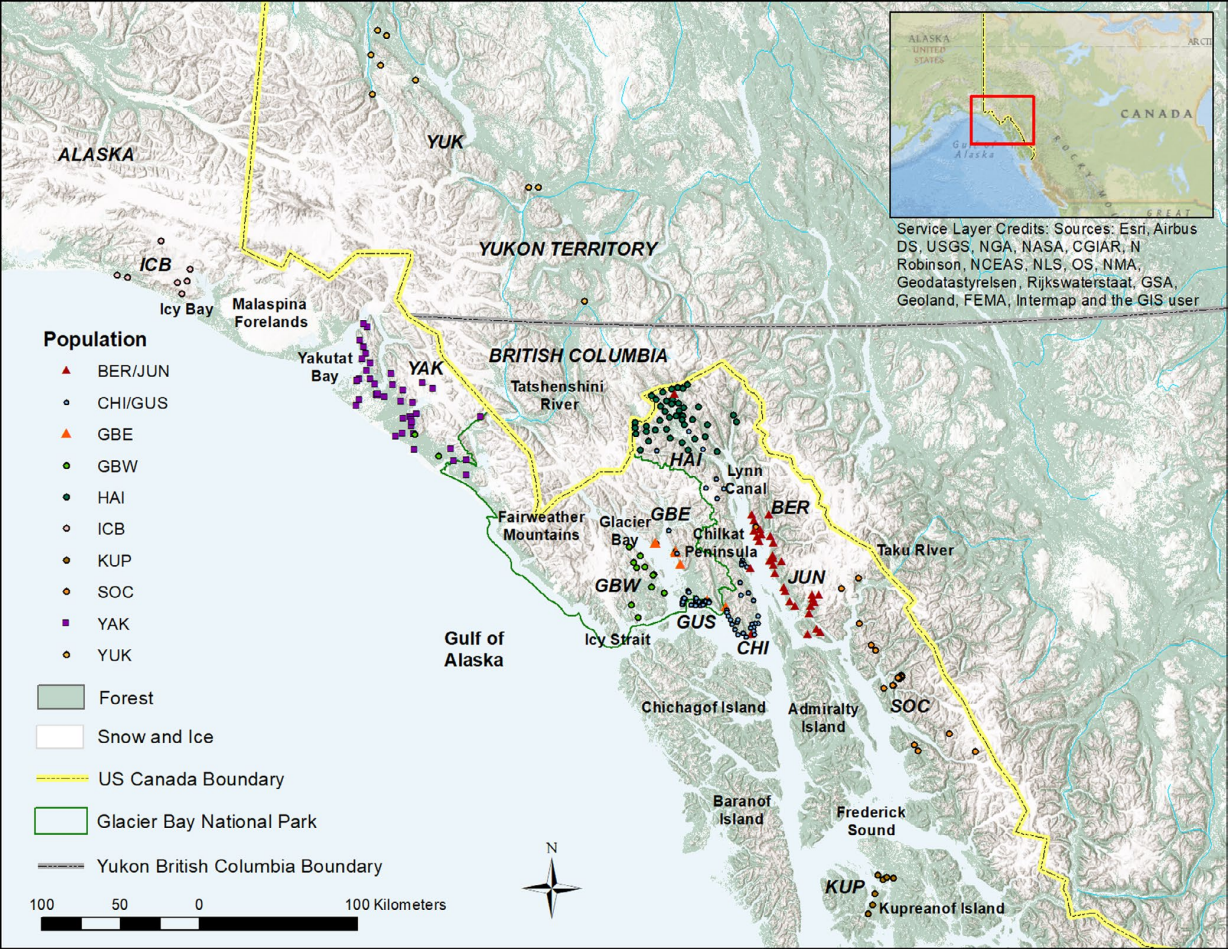
Population assignments by sampling locations of 284 black bears identified using Program STRUCTURE and *K* = 10 in Southeast Alaska and Yukon Territory, 2002–2014. Populations are as follows: Berners/Juneau (BER/JUN), Chilkat Peninsula/Gustavus (CHI/GUS), Glacier Bay East (GBE), Glacier Bay West (GBW), Haines (HAI), Icy Bay (ICB), Kupreanof Island (KUP), South Coast Mainland (SOC), Yakutat (YAK), and Yukon (YUK)

At STRUCTURE *K* = 10 only two bears were sampled on one side of Lynn Canal (sampled in Chilkat sampling area on west side) but assigned to a population on the opposite side (Juneau/Berners on east side; Figure 6). These individuals were both identified from harvest samples in which the hunters identify the location of the harvest. One of these hunters has previously falsified harvest locations, whereas the other is believed to be credible (A. Crupi, personal communication). Translocations may be the source of some migrants as at least one black bear involved in human–bear conflicts was moved from Juneau to the Chilkat peninsula in the 1990s (N. Barten, personal communication). No bears sampled on one side of Glacier Bay were assigned to a population from the other side. Similarly, there is no evidence of migration between Icy Bay and Yakutat despite being adjacent but on opposite sides of Yakutat Bay. Frederick Sound also appeared to pose a barrier to genetic connectivity based on the lack of migrants between South Coast and Kupreanof populations, although sample size in Kupreanof was low (*n* = 7).

Peacock et al. (2007) found black bears in the Yakutat area to be the most genetically distinct in SEAK and suggested that the Fairweather Mountains were the barriers separating Yakutat from Chilkat; however, that study did not include samples from Glacier Bay National Park which lies directly between the two areas. Two additional populations of black bears (GBW and GBE) were found between Yakutat and the Chilkat Peninsula when noninvasive hair sampling was used to fill in the large geographic gap occupied by the national park. *F*_ST_ and Jost *D* values were greater between populations on opposite sides of Glacier Bay fjord (GBW vs. GBE, *F*_ST_ = 0.090) than populations on opposite sides of the Fairweather Mountains (Yakutat vs. GBW, *F*_ST_ = 0.061; Table 1). These results suggest that the fjord is a greater barrier than the ice‐covered mountains, likely because a low elevation coastal route is available at the base of the mountains. However, our results support evidence that ice‐covered mountains do pose genetic barriers, which could indicate increases in future genetic connectivity as glaciers rapidly shrink with climate change. Only three individual bears were assigned to a population on opposite sides of the ice field from their sampling site, including two bears from Berners assigned to South Coast. In both instances, a nonglaciated coastal route between the sampling regions was potentially available. In addition to high differentiation between populations on opposite sides of Glacier Bay and Lynn Canal fjords, *F*_ST_ and Jost *D* values were highest between populations that were farthest apart (Icy Bay and Kupreanof) and lowest between adjacent populations (Chilkat/Gustavus and Haines) supporting the Mantel test isolation by distance results. Exceptions to this pattern include the low differentiation found between South Coast and Yukon, despite large geographic distances (<450 km) indicating a possible mainland interior corridor of genetic connectivity between the two populations. Pelletier et al. (2012) found isolation by distance and low levels of differentiation between populations of black bears across a large (up to 550 km) contiguous landscape in Ontario, Canada. Although the landscape in our study area is highly fragmented with glaciers and fjords, the eastern portion borders more contiguous areas of Yukon Territory and BC which may serve as a corridor between coastal populations. Low sample size, particularly for the Yukon samples (*n* = 12) may also be a factor in these results. Similarly, the proportion of ancestry of bears sampled on Kupreanof that were assigned to the Icy Bay population (0.267; Table 1) indicates either an interior travel corridor, an unsampled population, or a spurious result based on low sample size of both sampling regions (both *n* = 7). Peacock et al. (2007) found black bears from Kupreanof (*n* = 34) clustered strongly with other island populations with very low proportion of assignments to mainland populations based on both nuclear and mitochondrial DNA. This previous study did not include samples from Icy Bay, so it is difficult to make direct comparisons, but provides further evidence that the Kupreanof–Icy Bay relationship found in our analysis may be a spurious result. Further evidence includes results of significant isolation by distance and high *F*_ST_ (0.174) and Jost's *D* (0.668) values between the two populations.

Another potential barrier for black bear dispersal is nonforested landscape such as the recently deglaciated northern shoreline of Glacier Bay. Black bears rarely occur in this region due to the recent deglaciation and lack of conifer or deciduous forest cover to which this species strongly associates (Lewis, 2012). As glaciers continue to recede, postglacial plant succession continues, and forest develops in northern Glacier Bay, we would imagine that GBW and GBE populations of black bears would expand their range northward and come into contact with each other more frequently and potentially mix. Lewis et al. (2015) proposed the northern portion of Glacier Bay represented a contemporary population‐level contact zone for brown bears that recolonized the coast from different populations on each side of the fjord and then funneled northward to mix in the northern regions with little admixture between populations. In the current study, one interesting area of overlapping populations was found in the land dividing the west and east arms of Glacier Bay where five bears were assigned to the GBE population and one was assigned to the GBW population. This area may provide opportunities for admixture between populations from the east and west side of Glacier Bay and thus a potential contact zone. Lewis et al. (2015) found evidence of a unique population of brown bears sampled predominantly on the shoreline of northern Glacier Bay, possibly derived from a historical colonizing population that has undergone genetic drift. At STRUCTURE *K* = 10 a similarly unique population of black bears (GBE) was found on the southeast side of Glacier Bay ranging into Gustavus and Chilkat sampling regions. Five black bears sampled in GBE were assigned to the Chilkat/Gustavus population, and two individuals assigned to the GBE population were sampled in Chilkat and Gustavus sampling regions, indicating that the two distinct populations overlap in range on the southeast shoreline of Glacier Bay. Similar to results from Lewis et al. (2015) with brown bears, distinct genetic clusters of black bears that overlap geographically may indicate that colonization of the shoreline of Glacier Bay from neighboring populations is recent and likely ongoing as glaciers continue to recede and plant communities develop from meadow to forest.

Female home‐range estimates derived from this GPS collar study were similar to others derived from very high‐frequency (VHF) collars to estimate black bear space use in SEAK (Erickson, Hanson, & Brueggeman, 1982; Robus & Carney, 1996), though substantially smaller than noncoastal populations in less fragmented landscapes at more southern latitudes (Pacas & Paquet, 1994; Vander Heyden & Meslow, 1999). The results of GPS collar data show small female home‐range size, likely shaped by the extensive barriers to movement, moderate densities, and abundant resources. It is important to note that the home‐range sizes derived in this study represent only a small portion of the study area and variability in home‐range sizes across the region is likely high as is variation in fragmentation and resource quality. Unfortunately, we were not able to learn more about male home‐range size in this region due to low sample size, as this would have greater implications on connectivity due to male‐mediated gene flow in the species.

Glacier Bay fjord and the ice‐covered mountains north of Glacier Bay appear to be a dividing point between the brown color morph of black bears in SEAK, which may have implications on subspecies designations although further genetic work is necessary. Only one of the 71 (1.4%) bears sampled west of Glacier Bay (GBW and Yakutat sampling regions) was brown colored compared to 31 bears (14.6%) sampled east of Glacier Bay (Figure 2). Glacier Bay and Lynn Canal may represent dividing points for the glacier morph but interestingly glacier bears were sampled on the west side of Glacier Bay but not the east, and on the east and north side of Lynn Canal, but not on the west. The genetic results corroborate the sighting data (Lewis et al., 2020) indicating that glacier bears are most common west of Glacier Bay, north and east of Lynn Canal, but largely absent from east Glacier Bay and the Chilkat Peninsula. Glacier bears were not sampled in the two populations between Glacier Bay and Lynn Canal: GBE and Chilkat/Gustavus. Sample size may have been a limiting factor for GBE (*n* = 12) but Chilkat/Gustavus had the largest sample size of any group (*n* = 81), so it is unlikely that glacier bears were missed accidentally in this population. The highest percentage of glacier bears (28%) were found in the Yakutat population. The frequencies of samples do not represent the proportion of glacier bears in each population due to differences in hunting and sampling effort in different regions. GBW is likely underrepresented due to the lack of harvest samples from within the park. *F*_ST_ and Jost *D* values do not indicate that black bear populations containing glacier bears are more closely related to each other; then, they are to those without glacier bears (Table 1). A possible explanation for the lack of relatedness among these populations is a common ancestral clade, and/or the presence of an unsampled population between Haines and Yakutat along the Tatshenshini River. Sightings records show multiple glacier bears in this region (Lewis et al., 2020). Another possible explanation is an association between glacier bears and large ice fields, which occur on the east side of Lynn Canal and the west side of Glacier Bay, but not between. This association would suggest a selective advantage for glacier bears in glacial environments.

Selective advantage has been proposed for the white pelage of black bears on the coast of BC known as the Kermode Bear (Reimchen & Klinka, 2017). Logging threats on Princess Royal Island in the 1990s led to research on the population structure of and genetic basis for *Ursus americanus kermodei* (Kermode Scientific Panel, 2007). “Kermodism” may have developed during the ice ages of the Pleistocene or may be a more recent mutation stemming from small populations undergoing genetic drift, but is believed to be maintained by genetic isolation, reduced population sizes, and possibly selection and assortative mating (Hedrick & Ritland, 2012; Marshall & Ritland, 2002). To explore possible contemporary fitness benefits of the white color morph, Klinka and Reimchen (2009) compared salmon foraging behavior and found greater fishing success by Kermode bears than black morphs during daylight hours. These results combined with stable isotope evidence of increased use of marine resources by Kermode bears indicate a possible adaptive advantageous ecological niche of the Kermode bears that may facilitate the persistence of the color morph (Reimchen & Klinka, 2017). Further genetic work is needed to determine the genetic basis for the glacier color morph as well as possible evidence of selective advantage in peri‐glacial environments.

## 5 CONCLUSIONS

Marine fjords limit genetic connectivity of black bears as do glacier‐covered mountains and nonforested regions unless there are low elevation forested routes around such features. Glacier Bay fjord appears to be a dividing point between black bear populations with brown color morphs and those without in SEAK, which may have implications on subspecies designations although further genetic testing is needed. Within the range of glacier bears, we found four populations of black bears contained the rare glacier pelage type and two populations that did not. Lack of geographic continuity and genetic relatedness between black bear populations containing glacier bears suggest a possible unsampled population in northwest BC that should be explored with additional samples if possible. Analysis of mitochondrial DNA may help determine if ancestral clades and possibly subspecies differences explain the geographic pattern of the color phase. Another possible explanation is an association between glacier bears and large ice fields, which would suggest a selective advantage for glacier bears in glacial environments. Such an association would increase the conservation risk of the color morph as glaciers decrease in size, so further investigation is needed to determine the adaptive and evolutionary significance of the glacier bear color morph. Determining the genetic basis of the glacier bear color morph will also be necessary to determine the frequency of the gene(s) across black bear populations containing the rare phenotype. These results would shed light on the rareness of the color morph across the glacier bear range and help focus conservation efforts to maximize and preserve genetic diversity of black bears as glaciation of the region decreases with climate change.

## CONFLICT OF INTEREST

The authors of this manuscript have no competing interests, financial or otherwise.

## AUTHOR CONTRIBUTIONS

**Tania Lewis:** Conceptualization (lead); data curation (lead); formal analysis (supporting); funding acquisition (lead); investigation (lead); methodology (equal); project administration (lead); resources (lead); software (supporting); supervision (lead); validation (equal); visualization (equal); writing – original draft (lead); writing – review & editing (lead). **Gretchen Roffler:** Conceptualization (supporting); data curation (equal); formal analysis (lead); investigation (supporting); methodology (supporting); software (lead); validation (equal); writing – original draft (supporting); writing – review & editing (supporting). **Anthony Crupi:** Conceptualization (supporting); data curation (equal); formal analysis (supporting); funding acquisition (supporting); investigation (supporting); methodology (supporting); project administration (supporting); resources (supporting); software (supporting); validation (equal); writing – original draft (supporting); writing – review & editing (supporting). **Ramona Maraj:** Data curation (supporting); formal analysis (supporting); investigation (supporting); methodology (supporting); resources (supporting); validation (supporting); writing – original draft (supporting); writing – review & editing (supporting). **Neil Barten:** Conceptualization (supporting); data curation (supporting); investigation (supporting); resources (supporting); writing – original draft (supporting); writing – review & editing (supporting).

## Supporting information

Table S1‐S3

## Data Availability

Microsatellite genotypes and sampling locations are accessible: https://orcid.org/0000‐0001‐5687‐3722 with this link: https://datadryad.org/stash/share/poaie2BfxL8VHAD7tE7s3y2rZGadiLLMna_fCJ_BcWI.
