# Supplementary material for: Unraveling the mystery of the glacier bear: Genetic population structure of black bears (Ursus americanus) within the range of a rare pelage type
Source: Ecol Evol. 2020 Jun 27;10(14):7654–68. doi: 10.1002/ece3.6490 (PMC7391538; doi:10.1002/ece3.6490)
Supplement: Supplementary file 1 — Table S1‐S3 [file ECE3-10-7654-s001.pdf]

Lewis et al. Supplemental

T1. Black bear (*Ursus americanus*) home range estimates in northern Southeast Alaska, 2003–2015. Home ranges calculated using fixed kernel density estimation with least-squares cross-validation smoothing parameter.

Sex	Number of bears	Number of GPS locations ($\bar{X}$ ± SD)	Number of days collar deployed ($\bar{X}$ ± SD)	Home range size (km^2) ($\bar{X}$ ± SD)
Female	11	3,410.3 ± 2,398.5	351.3 ± 204.4	13.0 ± 7.4
Male	1	11,737	389	86.9

T2. Sample size, mean number of alleles per locus (*A*), private alleles (*PA*), allelic richness (*AR*), observed (H_o) and expected (H_e) heterozygosity, and inbreeding coefficient (F_{IS}) for each sampling region in Southeast Alaska and Yukon Territory, 2002–2014.

Sampling Region	Code	n	*A*	*PA*	*AR*	H_e	H_o	F_{IS}
Berners Bay	BER	17	5.48	0	4.46	0.688	0.717	-0.011
Chilkat Peninsula	CHI	32	5.86	1	4.18	0.652	0.621	0.065
Glacier Bay East	GBE	16	4.95	1	4.13	0.658	0.652	0.041
Glacier Bay West	GBW	18	4.52	3	3.92	0.633	0.688	-0.058
Gustavus	GUS	42	5.19	4	3.92	0.642	0.657	-0.011
Haines	HAI	48	6.62	2	4.44	0.685	0.678	0.020
Icy Bay	ICB	7	3.29	6	3.29	0.556	0.565	0.062
Juneau	JUN	15	5.86	2	4.84	0.723	0.740	0.011
Kupreanof	KUP	7	3.71	6	3.71	0.572	0.605	0.018
South Coast	SOC	18	6.71	5	5.29	0.762	0.732	0.069
Yakutat	YAK	52	5.48	1	3.71	0.590	0.586	0.017
Yukon	YUK	12	5.95	4	5.19	0.741	0.798	0.010

T3. Pairwise comparison of F_{ST} (below the diagonal) and Jost's D values (above the diagonal) from analyses among sampling regions in Southeast Alaska and Yukon Territory, 2002–2014. All values were significant at p > 0.01. Populations are as follows: Berners/Juneau (BER/JUN), Chilkat Peninsula/Gustavus (CHI/GUS), Glacier Bay East (GBE), Glacier Bay West (GBW), Haines (HAI), Icy Bay (ICB), Kupreanof Island (KUP), South Coast Mainland (SOC), Yakutat (YAK), and Yukon (YUK).

	BER	CHI	GBE	GBW	GUS	HAI	ICB	JUN	KUP	SOC	YAK	YUK
BER		0.312	0.324	0.379	0.332	0.275	0.493	0.039	0.515	0.249	0.364	0.346
CHI	0.126		0.069	0.160	0.030	0.072	0.465	0.293	0.515	0.412	0.163	0.302
GBE	0.125	0.033		0.189	0.086	0.126	0.425	0.308	0.496	0.370	0.225	0.319
GBW	0.152	0.076	0.087		0.178	0.176	0.488	0.339	0.484	0.373	0.117	0.248
GUS	0.138	0.016	0.042	0.087		0.109	0.458	0.324	0.552	0.428	0.188	0.319
HAI	0.106	0.033	0.054	0.077	0.051		0.414	0.244	0.479	0.352	0.215	0.252
ICB	0.199	0.206	0.187	0.223	0.210	0.175		0.450	0.639	0.544	0.499	0.380
JUN	0.015	0.111	0.110	0.128	0.127	0.089	0.171		0.469	0.182	0.347	0.254
KUP	0.202	0.219	0.208	0.217	0.238	0.193	0.290	0.172		0.486	0.592	0.550
SOC	0.078	0.139	0.118	0.127	0.150	0.114	0.182	0.052	0.161		0.448	0.186
YAK	0.169	0.089	0.118	0.067	0.102	0.107	0.252	0.155	0.281	0.179		0.260
YUK	0.109	0.110	0.108	0.094	0.121	0.088	0.141	0.074	0.188	0.050	0.118	
